# Simulation and Training of Gynaecological Skills

**Published:** 2018-03

**Authors:** H Ferreira, Y van Belle, V Tanos, B Rabischong, G Grimbizis, A Di Spiezio Sardo, R Campo

**Affiliations:** Gynecology Minimally Invasive Surgery Unit, Centro Hospitalar Universitário do Porto, 4099 Porto, Portugal; European Academy of Gynaecological Surgery, 3000 Leuven, Belgium; Aretaeion Medical Center, 2024 Nicosia, Cyprus.; Department of Gynecological Surgery, CHU Estaing Clermont-Ferrand, 63000 Clermont- Ferrand, France; First Department Obstetrics/Gynecology, Aristotle University of Thessaloniki, 54124 Thessaloniki, Greece; Department of Public Health, University of Naples Federico II, Via S. Pansini 5, 80131 Naples, Italy

**Keywords:** Gynaecological skills, Endoscopic surgery, Medical Simulation, GESEA Diploma Programme

## Abstract

In order to offer our patients, the “state of the art” treatment in gynaecology, we need a structured teaching program for trainees concerning the gynaecological skills. In recent years, training and education in endoscopic surgery has been critically reviewed. Clinicians, both surgeons as gynaecologists who perform endoscopic surgery without proper training of the specific psychomotor skills, are at higher risk to increased patient morbidity and mortality. The traditional apprentice-tutor model is no longer valid for developing all skills necessary in gynaecological surgery, particularly in endoscopy. Endoscopic training should happen at both the theoretical and the practical skill level. The acquisition of the correct knowledge regarding general laparoscopy, hysteroscopy and standard level procedures is as important as learning the necessary psychomotor skills to successfully perform endoscopic manipulations. Training in the operating room can only start when it is proven that knowledge and skills are present.

To learn and train total abdominal hysterectomy by laparotomy there are inexpensive simple models that can be used, which are easy to reproduce. The development, construction, cost, and utility of a low-cost and anatomically representative vaginal hysterectomy simulator also has been described.

The complexity of modern surgery has increased the demands and challenges to surgical education and the quality control.

## Introduction

Today it is generally accepted that the traditional apprentice-tutor model is no longer valid for training all skills necessary in Gynaecological surgery and more specifically endoscopic surgery (Campo et al., 2016b). This agreement is based on the recognition that, in contrast to open surgery, endoscopic surgery demands surgical skills and psychomotor skills that should not necessarily be trained simultaneously. Increasing evidence strongly suggests that psychomotor skills must be trained earlier and outside the operating room, and several models have been proposed for this aim (Sroka et al., 2010; Diesen et al., 2011; Munro, 2012; Mulla et al., 2012; Escamirosa et al., 2015; Hofstad et al., 2013).

Consequently, six leading professional organisations in gynaecology, the ESGE, EBCOG, EAGS, ENTOG, ACOG and AAGL, have issued a joint recommendation regarding endoscopic surgical training and quality assurance. This recommendation states that each hospital teaching endoscopic surgery should provide an endoscopic dry lab for training and improving the physician’s proficiency in endoscopic surgery skills.

The rationale behind this recommendation is that endoscopy demands both psychomotor and surgical skills. It is vital that these psychomotor skills are trained and tested in a safe environment prior to training in the operating room, as it reduces patients’ morbidity and mortality rates, it reduces the patients’ exposure to unskilled trainees and it greatly increases educational efficiency.

In this section, examples of training simulators are provided, some of which are mentioned by commercial name or brand. Please note that many different training tools are available and that the field of simulation is continuously developing. Recommendations and advice on specific training simulators or tools may be found at ESGE.

Knowledge acquisition and psychomotor skills training need to happen via a structured approach, which only allows trainees to progress in the programme when clearly defined and measurable goals have been attained and assessed. A recent study shows that training of basic laparoscopic psychomotor skills (hand-eye coordination) prior to training of suturing skills improves both the acquisition and the retention of more advanced laparoscopic tasks, such as laparoscopic intra-corporeal knot tying (Molinas et al., 2017), which further indicates the need for a structured approach in which basic psychomotor skills are trained prior to more advanced laparoscopic skills.

## Training curriculum

Training trainees to become proficient gynaecological surgeons should not start with training in the OR, but instead in a simulation setting (Molinas et al., 2017). A structured gynaecological training program should fully embrace this approach and encompass a series of well-defined steps, combining dry lab training with in-OR training. At each phase, an assessment should take place to validate whether the trainee can proceed to the next level.

The following steps can be defined in this approach:

- Basic endoscopic training (dry lab): Knowledge acquisition of general endoscopic principles and techniques combined with basic practical endoscopic skill training.
- Start of in-OR training: After the trainee has proven to be in possession of the necessary basic endoscopic knowledge and practical skills, the in-OR training can be started. In this phase, the trainee can assist an expert endoscopist mentor and is exposed to basic OR practices and teamwork.
- Advanced endoscopic training (dry lab): Knowledge acquisition of standard level procedures and training of advanced practical skills.
- Start of in-OR surgery: The skills laboratory phase is past and live surgery may be undertaken, according to a stepwise approach starting with close supervision and simple procedures and step by step expanding to less supervision for simple procedures and moving on to more complex ones.

**Figure:**
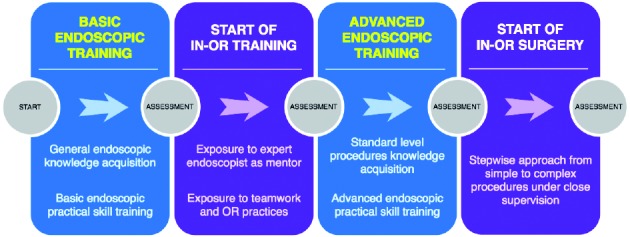


This approach aims to train and assess the necessary endoscopic skills in a dry lab setting as much as possible, before moving onto live patients in the OR. The benefit of this is threefold:

1. Trainees are much more secure when they enter the OR as they are more confident that they have acquired the necessary knowledge and skills;
2. Expert mentors do not loose time teaching basic skills, can receive proper assistance from trainees and can focus more on the procedures at hand;
3. The patient receives better care due to proper trained trainees and is much less exposed to unskilled trainees.

Endoscopic training should happen at both the theoretical and the practical skill level. The acquisition of the correct knowledge regarding general laparoscopy and standard level procedures is as important as learning the necessary psychomotor skills to successfully perform endoscopic manipulations. Training in the operating room can only start when it is proven that knowledge and skills are present.

## Knowledge acquisition

The acquisition of general endoscopic knowledge and, at a later stage, of standard level procedures can happen prior to or in parallel with practical skill training. To make this type of training as affordable as possible, it can be offered as an easily accessible structured e-learning programme in combination with self-evaluation modules which the trainee completes and his or her own pace.

This knowledge acquisition can be further supported by one or more group-based sessions and should always be followed by an official assessment of the trainee.

Example: The Winners Project e-learning programme offers free of charge online tutorials and theoretical assessment of laparoscopic and hysteroscopic knowledge.

## Practical skills training

As it is widely accepted that dry lab training prior to training in the OR reduces patients’ morbidity and mortality in all endoscopic surgical disciplines, it is critical that practical skills can be trained in-house by using a structured approach with validated methods and training tools. Furthermore, a management platform is required to facilitate this structured approach regarding data collection and report generation.

## Management platform

To achieve a structured programme, it is vital that the necessary support software is available that can manage all data collection. This collection of data occurs at two levels: the profiles and experience of the trainees, and the results of the training and test sessions that are carried out. Ideally, such a system should offer the possibility to view the progress of the trainees over time, to compare the training results with peers by means of a benchmark according to exposure level and to automatically issue all relevant reports and potential certificates.

## Examples & equipment

### Laparotomy and basic surgical skills

For basic skills, very simple models are described to train the surgical skills:

- foam rubber tube, which can be used to train e.g. suturing skills or manufacture a simple vaginal cuff model;
- tubular balloons that can replicate the ureters (simulate ureter anastomosis, etc) and the tubes (tubal surgery)
- Cloths for suturing and knotting training
- Balloons to replicate ovarian cystectomy

To learn and train total abdominal hysterectomy by laparotomy there are inexpensive simple models that can be used, which are easy to reproduce (Hong et al. 2012). When combined with a didactic lecture and real instruments for instruction, it may be a valuable simulator tool in trainee education. The total abdominal hysterectomy model was constructed to include the uterus, uterine arteries, ovaries, bladder, supporting ligaments, triple pedicle, vagina, and ureters.

### Vaginal hysterectomy (VH), posterior and anterior compartments repair

The development, construction, cost, and utility of an inexpensive and anatomically representative VH simulator has been described. After multiple materials and solutions were evaluated and tested, a VH and posterior/anterior compartments repair simulators that incorporated strengths from each concept has been constructed (Barrier et al. 2012) (Geoffrion et al. 2016).

## laparoscopy

The advised dry lab setup for training laparoscopic skills includes the following:

- Pelvic trainer: The pelvic trainer simulates the ports that are used when performing a laparoscopic procedure on a patient. Their main goal is to restrict the movement of the instruments and to necessitate the use of an endoscopic camera. Together with training models, they form an ideal combination to train skills like camera navigation and proper hand-eye coordination.
- Training models for laparoscopic psychomotor skills: The training model is placed inside in a pelvic trainer and provides a number of exercises which will train the basic laparoscopic instrument handling skills, like camera navigation and hand-eye coordination, including bimanual coordination.
- Training models for laparoscopic suturing skills: The laparoscopic suturing skills model is placed inside a pelvic trainer and allows to perform stitching and knotting exercises and various levels of difficulty.
- All-in training model for laparoscopic psychomotor and suturing skills example: Encilap all-in training model

Using a tubular balloon, it is possible to make a longitudinal incision to replicate the salpingostomy, and a complete cut for salpingectomy.

Using a balloon filled with water involved by a second balloon, it is possible to make the translation of an ovarian cystectomy (removing the external balloon avoiding the rupture of the internal one).

**Figure 2:**
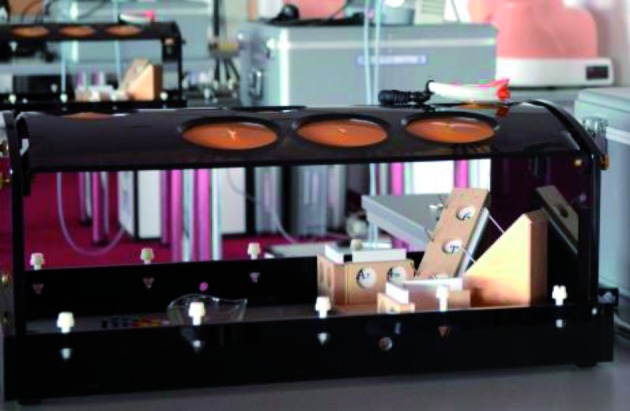
— Example: LASTT Model placed inside pelvic trainer. LASTT (Laparoscopic Skills Training and Testing Method) Training Model. The LASTT model is a validated training model that represents the spatial distribution and orientation of the different planes and angles of a female pelvis. It provides validated laparoscopic exercises to train and test the individual on their laparoscopic psychomotor skills (Campo et al. 2016b) (Campo et al. 2016b) (European Society for Gynaecological Endoscopy. and ProQuest Central. 2004) (Campo et al. 2012) (Palter et al. 2013) (Gala et al. 2013) (Burden, Oestergaard, and Larsen 2011) (Antoniou et al. 2012) (Hur et al. 2011) (Antoniou et al. 2012) (He et al. 2013) (Wang et al. 2016) (Nieboer et al. 2009) (Medeiros et al. 2009) (De Win et al. 2015) (Sinitsky, Fernando, and Berlingieri 2012) (Ascher-Walsh and Capes 2007) (Gala et al. 2013) (Ghomi et al. 2007) (Stefanidis, Acker, and Heniford 2008)

**Figure 3:**
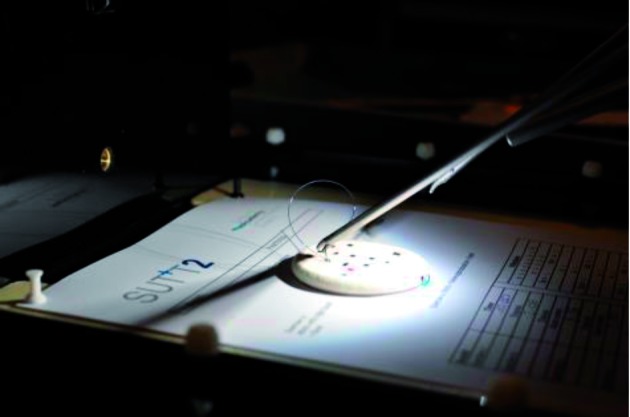
— Example: SUTT2 Model placed inside pelvic trainer. SUTT (Suturing and Knot Tying Training and Testing Method) Training Model The SUTT model consists of two levels, SUTT1 and SUTT2 with increasing levels of difficulty and trains laparoscopic suturing and knot tying. The exercises are performed with standard laparoscopic instruments: 10 mm 0o optic and needle holders. SUTT1 consists of one exercise where stitching and knot tying is evaluated. SUTT2 consists of four exercises that address Greek suturing, stitching with both hands, knot tying and tissue approximation..

**Figure:**
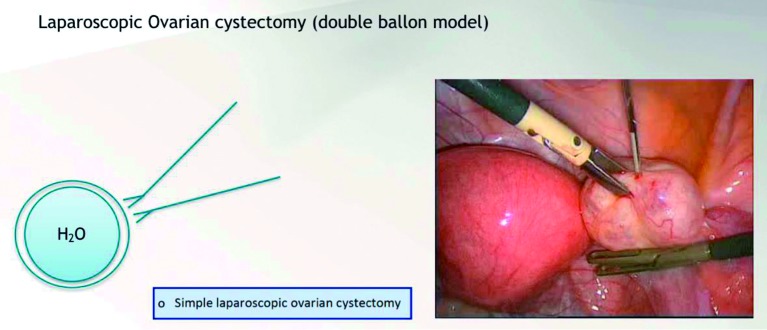


## hysteroscopy

The advised dry lab setup for training hysteroscopic skills includes the following:

- Female genital model To make the simulation as close to real life as possible, it is advisable to use female genital models in which the training models can be placed.
- Training models for hysteroscopic psychomotor skills The training model is placed inside the female genital model and provides relevant exercises which simulate all possible movements during a hysteroscopic procedure. Example:

**Inside view of HYSTT Exercise 1 and 2.**
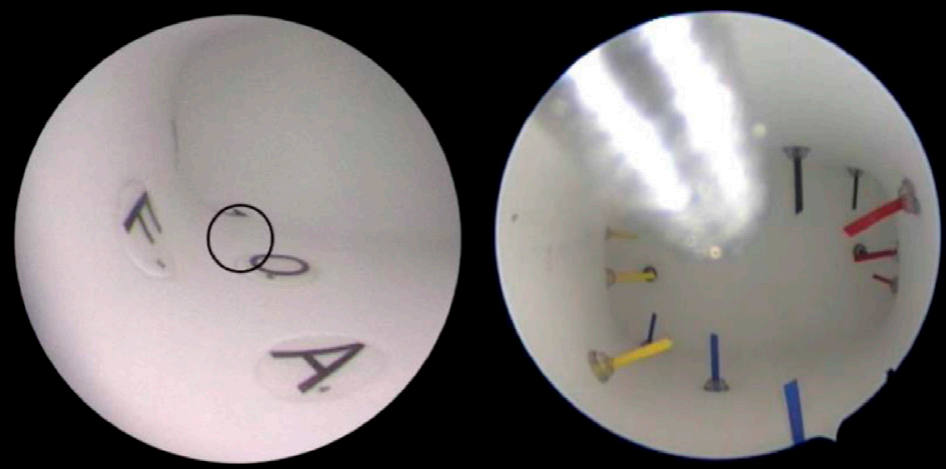
HYSTT (Hysteroscopic Skills Training and Testing Method) Training Model The HYSTT model tests and trains hysteroscopic camera navigation and instrument handling. The model is made in a shape similar to a human uterus and installed in female genital model. It has two levels of difficulty, HYSTT1 and HYSTT2 and both levels consist of two exercises. Exercise 1 evaluates the skills of an individual to handle the camera and work with a 30° optic in an hysteroscopic environment. Various sets of modules are used in order to eliminate the memory effect for the participants. Exercise 2 evaluates the skills of simultaneous camera and instrument handling and hand-eye coordination skills as its goal is to pick and extract 14 pins.

- Training models for diagnosis Training of diagnostic cases is typically done by means of lectures and videos. However, using simulated models offers a significant benefit over traditional methods as they teach the trainee how to enter the cavity and proceed with caution, to identify the anatomical position of the abnormalities, to properly formulate what can be seen, to present this to their peers and to stipulate a resulting diagnosis. The diagnostic training models are placed inside a female genital model.

### Gynaecological skills to be trained (core curriculum)

Skills in outpatient clinic:

- Placement of Intra Uterine Device
- Placement of subcutaneous implants
- Endometrial biopsy
- Colposcopy (with biopsy)
- LLETZ of the cervix

Basic (core) conventional surgical skills:

- Punch biopsy under local anaesthesia
- Surgical marsupialization of cyst
- Surgical excision of abscess

More advanced (core) conventional surgical skills

- Laparotomy with minimal adhesiolysis
- Salpingo-oophorectomy by laparotomy
- Simple anterior vaginal repair
- imple posterior vaginal repair
- Myomectomy of subserous myoma by laparotomy
- Colpocleisis (at least in simulation setting)

Basic (core) endoscopic skills:

Laparoscopy:

- Diagnostic laparoscopy
- Diagnostic laparoscopy with tubal testing
- Simple laparoscopic adhesiolysis
- Laparoscopic sterilization
- Laparoscopic removal of ectopic pregnancy (salpingostomy) or salpingectomy
- Laparoscopic needle aspiration of simple cysts
- Laparoscopic electrocoagulation of the ovary
- Simple laparoscopic ovarian cystectomy
- Laparoscopic salpingo-oophorectomy

Hysteroscopy:

- Diagnostic hysteroscopy
- Diagnostic hysteroscopy with tubal testing
- Hysteroscopic polyp resection
- Hysteroscopic myoma resection type 0-1 (< 4 cm)

### Integrating simulation training in the curriculum

According to Harden et al. (2016) and Thomas et al. (2016):

- Needs: implement gynaecological skills training in prior to clinical activities
- Aims and objectives: develop uniform and structured educational and training programs in Europe
- Content: structured educational, training and assessment program in gynaecology
- Organisation of content: Free access to peer reviewed tutorials with self-evaluated models; Acquiring psychomotor skills with dry lab inanimate models; Standardized validated evaluation of the above skills; Entering the clinical teaching program if appropriate.
- Educational strategies: Structuring from knowledge to skills, and then to clinical activity; Objective evaluation module for each of the above; Objective evaluation methodology of the tutors.
- Teaching methods: Accessible, validated and affordable; Standardized dry lab training exercises; Internet access; Apprentice-tutor model after theoretical knowledge and psychomotor skills acquisition.
- Assessment: objective structural exercises evaluation, certification, preferably external assessors, reports by tutors or supervisors, scales of self-assessment, continuous and end-of course assessment, exams for theoretical knowledge evaluation.
- Communication: local scientific societies, teaching hospitals committees, trainees, tutors/supervisors.
- Educational environment: Local infrastructure with access to knowledge (online video tutorials, structured learning programs) and dry labs; Larger skills labs for surgical competences with a dedicated training environment where you acquire inter-professional team skills, dedicated endoscopic clinical teaching facilities (animal lab, life surgery, virtual reality).
- Process: Inform national societies about the content of the program. Provide them different tools needed for applying the program: - Free access to peer reviewed tutorials with self-evaluated models (like GESEA program); - Acquiring psychomotor skills with dry lab inanimate models (pelvic trainers, boxes where would be possible to perform simple exercises with pins/rings using a webcam);
- Acquiring surgical competences using accessible and inexpensive models (e.g. abdominal and vaginal hysterectomy, ovarian cystectomy, ectopic pregnancy, anterior and posterior compartments defects correction) following a “step by step” simulation program.
- Providing standardized validated evaluation of the above skills (online score platforms); If appropriate entering into the clinical teaching program.

### Example of a simulation training program in endoscopic skills

The European Academy of Gynaecological Surgery, together with the ESGE, has elaborated a well-balanced diploma curriculum: Gynaecological Endoscopic Surgical Education and Assessment (GESEA) programme which is based on a structured approach similar to what is proposed in this document (Campo et al., 2016a).

The GESEA Diploma Programme currently consists of two active levels, a Bachelor level and a Minimal Invasive Gynaecological Surgeon (MIGS) level.

- The GESEA Bachelor (level 1) is open to all endoscopists and specifically aimed at trainees. It starts with the bachelor tutorials on the Winners E-learning platform and is followed by practical psychomotor skill exercises in for laparoscopy (LASTT and SUTT1) and hysteroscopy (HYSTT1). The exam consists of a theoretical test and practical tests and the obtained certificate serves as a license to enter the OR as the programme provides the necessary skills needed to start in-OR training.
- The GESEA MIGS (level 2) is open to both gynaecologists and reproductive surgeons and starts with more in-depth tutorials on the Winners E-learning platform. The training exercises feature advanced practical psychomotor skills (LASTT, SUTT2 and HYSTT2) and the obtained certificate serves as a license for conventional gynaecological laparoscopic and hysteroscopic surgical interventions.

To be following this program, one will require access to (minimum):

1. Inanimate models for open surgical skills
2. Endoscopic GESEA program
3. Infrastructure

**Figure:**
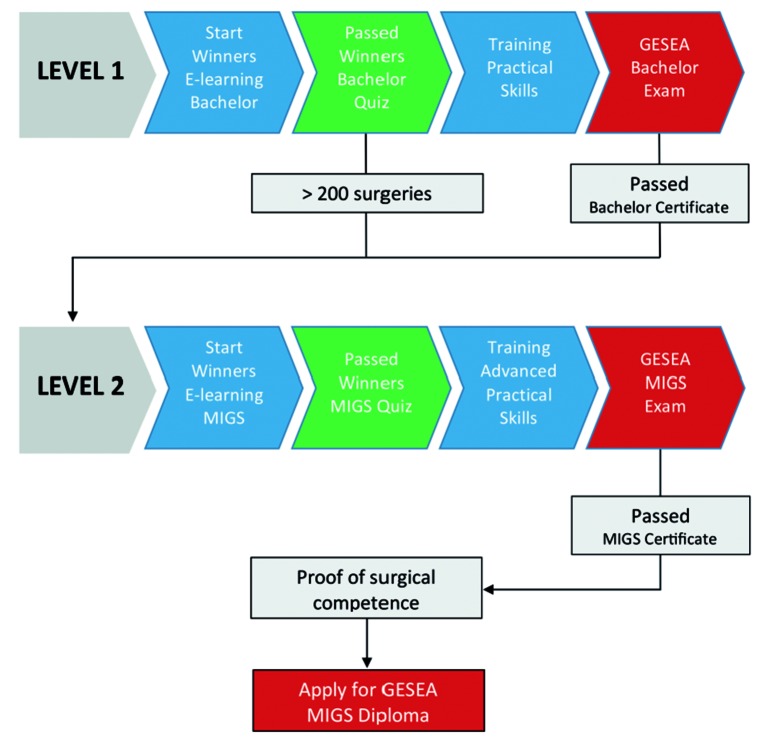


CORE: Dry Skill lab installed with computer access for online video tutorials, endoscopic hardware and training tools preferentially accessible to all surgical disciplines. Virtual reality trainers are acceptable but do not have an advantage above the low cost inanimate models.

ELECTIVES: Virtual reality trainer, inter-professional team skills, cadaver training and animal training courses. Live surgical sessions or streaming learning sessions. To profit from professional training environment it should be organised in regional or even supra-national training centres.

## Conclusion

The correct education and training on gynecological skills should be supported on tested and validated models allowing a uniform and effective implementation. This structured teaching program, supported on adequate simulation tools, may be diffused among trainees resulting at the end in an improvement on the health care offered to our patients.
